# Peptide–Membrane Interactions Monitored by Fluorescence Lifetime Imaging: A Study Case of Transportan 10

**DOI:** 10.1021/acs.langmuir.1c02392

**Published:** 2021-10-29

**Authors:** Sara Anselmo, Giuseppe Sancataldo, Hanne Mørck Nielsen, Vito Foderà, Valeria Vetri

**Affiliations:** † Dipartimento di Fisica e Chimica−Emilio Segré, Università degli Studi di Palermo, Viale delle Scienze ed. 18 90128, Palermo, Italy; ‡ Department of Pharmacy, University of Copenhagen, Universitetsparken 2 2100, Copenhagen, Denmark

## Abstract

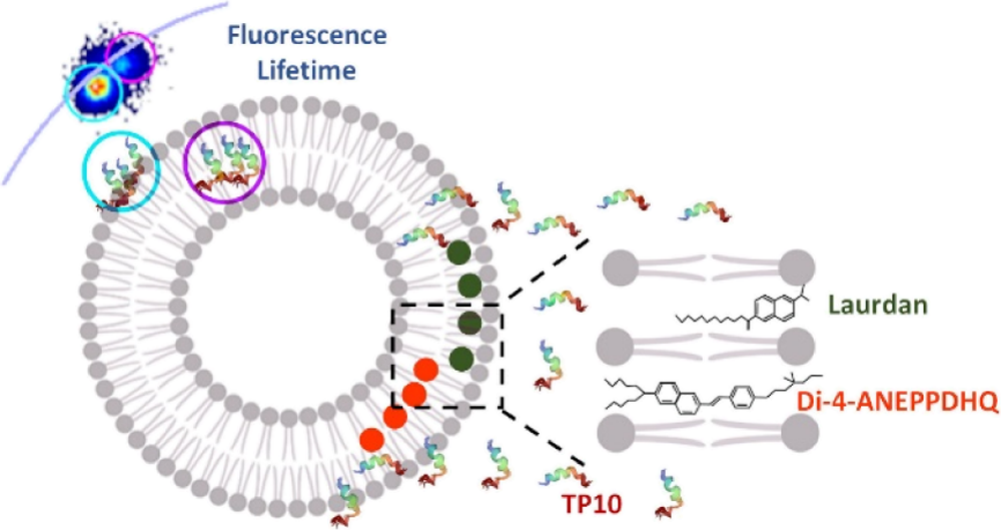

The interest on detailed analysis of peptide–membrane interactions is of great interest in both fundamental and applied sciences as these may relate to both functional and pathogenic events. Such interactions are highly dynamic and spatially heterogeneous, making the investigation of the associated phenomena highly complex. The specific properties of membranes and peptide structural details, together with environmental conditions, may determine different events at the membrane interface, which will drive the fate of the peptide–membrane system. Here, we use an experimental approach based on the combination of spectroscopy and fluorescence microscopy methods to characterize the interactions of the multifunctional amphiphilic peptide transportan 10 with model membranes. Our approach, based on the use of suitable fluorescence reporters, exploits the advantages of phasor plot analysis of fluorescence lifetime imaging microscopy measurements to highlight the molecular details of occurring membrane alterations in terms of rigidity and hydration. Simultaneously, it allows following dynamic events in real time without sample manipulation distinguishing, with high spatial resolution, whether the peptide is adsorbed to or inserted in the membrane.

## 1 Introduction

Peptide–membrane interactions are implicated in a broad range of biological processes, such as protein trafficking, cellular signaling, and ion channel formation, which are fundamental for the development of cellular functions.^1^ These interactions are also recognized to be the reason for toxicity in relation to amyloidosis.^2−4^ Under pathogenic conditions, amyloid oligomers can indeed accumulate at cell membrane interfaces causing changes to membrane integrity, structural organization, hydration, and/or conductance. These may in turn cause cell damages via unsolicited mechanisms such as alteration of ion homeostasis, deregulation of signal transduction, and changes in membrane trafficking.^5,6^

A large class of membrane-active peptides perform specific functions in living organisms’ defense or offense systems via actively targeting the lipid membrane of bacteria or other pathogenic agents.^7,8^ These peptides adsorb, fold, and form functional structures or aggregates inducing membrane modifications such as the formation of pores, membrane thinning, or breakage.^9−11^ The propensity of these peptides to aggregate at the membrane surface, even at low peptide/lipid ratios, suggests that an attractive, presumably lipid-mediated, force operates between them.^12^

Cell-penetrating peptides (CPPs) are a class of peptides able to cross cellular membranes and deliver macromolecular cargoes to the inside cells by nondestructive interactions and translocation mechanisms.^13^ The mechanisms include endocytosis, some do not involve specific receptors, some involve direct translocation, and for some CPPs the mechanism of action remains elusive.^9,10^ Irrespective of the mechanism, the initial step is the membrane interaction.

Membrane–peptide interaction may involve multiple complex mechanisms, which are strongly dependent on both the membrane lipid composition and structural details (charge, hydrophobicity, *etc.*) of the bioactive peptide chains, and may furthermore change depending on the peptide concentration, temperature, and pH.^14^ In this context, membranes may modulate the specific antimicrobial peptide activity,^14,15^ the insertion level of CPPs,^16,17^ and amyloid-related membrane toxicity.^18−22^ Moreover, cholesterol and sterols are indicated as enhancers of “the membrane order” as they modify lipid packing and the balance between electrostatic and hydrophobic interactions. For example, the effects of antimicrobial peptides and the insertion of CPPs are often reduced in cholesterol-rich membranes, such as those of eukaryotic cells, while amyloidogenic proteins or toxins have shown higher affinity for a cholesterol-rich environment.^10,14^

Here, we explore the interaction of transportan 10 (TP10) with synthetic giant vesicles (GVs) used as model membranes by means of time-resolved quantitative fluorescence microscopy methods. TP10 is a 21 residue, amphipathic, cationic CPP, which is known to translocate, alone or together with molecular cargoes, across the plasma membrane of living cells with enhanced affinity for cancer cells.^23^ It is also able to interact with bacteria or model membranes mimicking the bacterial bilayer composition acting as an antimicrobial peptide.^24^ Specifically, TP10 was found to have antimicrobial effects against several pathogens, and the peptide showed potent growth inhibition against *Staphylococcus aureus,* permeabilizing and killing the cells without damaging human ones. These specific properties certainly depend on its structural properties and on the local concentration at the membrane. TP10 contains a high proportion of positively charged amino acids (four lysines), no negative charges, and the N-terminus imparting it a formal +5 charge at neutral pH. This favors interactions, mediated by electrostatic forces, with negatively charged bacterial surfaces.

Various mechanisms of penetration and membrane modification of TP10 as well as other membrane-active peptides have been proposed to date, including pore formation and transient membrane modifications, which imply peptide conformational transitions.^25^ In cells, penetration is believed to involve both endocytosis and direct translocation^26^ as described above. *In vitro* in membrane models, TP10 is found to induce graded release of the contents of phospholipid large unilamellar vesicles^27^ with competing mechanisms often acting in parallel including toroidal, sinking raft, and carpet models.^28^ Confocal fluorescence microscopy on negatively charged giant unilamellar vesicles, constituted by DOPC and DOPG as phospholipids, highlighted pore formation and translocation phenomena showing labeled peptide passage into the lumen.^27^ The membrane composition,^29^ details of the specific environment, and peptide concentration^27^ define if and how TP10 translocates or modifies membranes as well as its potency. However, simultaneously evaluating the affinity of the peptides for membranes, their ability to self-associate, and the role of the water phases in the membrane represent an obstacle, which prevents the community from fully highlighting the molecular mechanisms on the basis of the membrane transport.

Here, we use fluorescence spectroscopy coupled with confocal and multiphoton microscopy to analyze, in real time and in a noninvasive mode, the physical modifications induced by TP10 on phosphatidylcholine/phosphatidylglycerol (POPC/POPG) GVs and that this is hindered by cholesterol presence. Membrane lipid composition has a crucial role in protein–membrane interactions and controls the membrane binding activity of protein molecules.^30,31^ Model membranes are often used in order to analyze specific features of these interactions, their composition can be easily tailored, and they regulate phase separation into liquid-disordered, solid-ordered, and liquid-ordered phases. This behavior is thought to exist in living cell membranes which exhibit analogous dynamic heterogeneities linked to specific functions. In this study, anionic POPG together with zwitterionic POPC was chosen to mimic the negatively charged bacterial membranes. POPC is the most common phospholipid found in cell membranes and POPG can be used to mimic anionic phospholipids which confer a negative charge to the membranes.^30^ Moreover, cholesterol was used, which is known to regulate membrane fluidity by interacting with phospholipids and sphingomyelin. It is not present in bacterial membranes, while it is found in the mammalian ones.^9^

To highlight membrane changes, we exploited the properties of two fluorescent membrane dyes: Laurdan and di-4-ANEPPDHQ. Both dyes display a spectral red shift in emission between the liquid-ordered and -disordered phases, as well as a shortening of the fluorescence lifetime. Furthermore, these dyes sense physicochemical aspects of the membranes at different length scales as they occupy diverse locations in the membranes and possess different charges.^32,33^ Using fluorescence lifetime imaging microscopy (FLIM), we were able to analyze events occurring at the membrane interface in terms of both TP10 and lipid bilayer physical states. Using the FLIM phasor approach employing Laurdan and di-4-ANEPPDHQ as fluorescent probes, we localized and quantified the evolution of changes in membrane order and hydration, also distinguishing peptide internalization from absorption.

Our findings indicate that TP10 adsorbs on the GV surface and, at sufficiently high concentration, it inserts into the membrane where two distinct lipid phases coexist. The insertion is correlated with the increase in rigidity of the lipid bilayer and to its dehydration. At first, TP10 induces modifications in the outer part of the membranes and then, depending on the balance between hydrophobic and electrostatic interactions, it propagates in the innermost regions experiencing environments with different structural organizations and distinct fluidity.

## 2 Materials and Methods

### 2.1 Materials

2-Oleoyl-1-palmitoyl-*sn*-glycero-3-phosphocholine (POPC-42773), 2-oleoyl-1-palmitoyl-*sn*-glycero-3-phospho-rac-(1-glycerol) sodium salt (POPG-63371), cholesterol (Chol-C8667), fluorescein (46955), 6-dodecanoyl-2-dimethylaminonaphthalene (Laurdan-40227), and dimethyl sulfoxide (DMSO-1029521000) were purchased from Sigma-Aldrich. Di-4-ANEPPDHQ (D36802) and Alexa Fluor 405 NHS ester (A30000) were purchased from Thermo Fisher Scientific, Waltham, Massachusetts. TP10 and TP10 labeled with carboxyfluorescein (CF) at the N-terminus (CF-TP10) were purchased from EZBiolab, Parsippany, New Jersey.

### 2.2 GV Preparation and Staining

GVs were prepared from POPC/POPG in a 1:2 molar ratio and POPC/POPG/Chol GVs in a 1:2:1 molar ratio. The lipid stocks were prepared by mixing the lipids in a 3:2 chloroform/methanol solution and dried overnight to form lipid films in round flasks on a rotary evaporator Buchi (Flawil, Switzerland), Rotavapor R-215, equipped with the Buchi Vacuum Controller V-855. The dry lipid films were hydrated using a 20 mM potassium phosphate buffer at pH 7 and sonicated for 5 min. The resulting sample comprised a heterogeneous distribution of multilamellar GVs with a diameter of several micrometers.

After the GV formation, the sample was diluted 1:10 and, when needed, labeled using Laurdan and di-4-ANEPPDHQ. Stock solutions of Laurdan (100 μM) and di-4-ANEPPDHQ (100 μM) were prepared in DMSO and stored protected from light exposure. Both dyes were added to diluted GVs in a probe–lipid molar ratio of 1:500 and left to equilibrate for 3 h before measurements.

### 2.3 Steady-State Fluorescence Emission Spectra

Fluorescence measurements were acquired at room temperature using a Jasco-FP-8500 spectrofluorometer equipped with a Jasco ETC-815 Peltier as the temperature controller in 1 cm path-length quartz cuvettes.

#### 2.3.1 CF Fluorescence Emission

CF-TP10 fluorescence emission spectra were measured before and after GV addition. The spectra were acquired, in the range 470–650 nm, using λ_exc_ = 480 nm with an excitation bandwidth of 2.5 nm, an emission bandwidth of 5 nm, a response time of 1 s, a data interval of 0.5 nm, and a scan speed of 100 nm/min.

### 2.4 Membrane Fluidity Measurements in Bulk

Laurdan fluorescence emission and, in particular, its generalized polarization (GP) were analyzed to evaluate the effect of TP10 on POPC/POPG and POPC/POPG/Chol membranes. Fluorescence emission spectra were acquired in the range 370–650 nm as a function of time every 5 min after peptide addition at two different concentrations (300 nM and 1.3 μM). The excitation wavelength was λ_exc_ = 380 nm and excitation and emission bandwidths were 5 nm, response time 1 s, data interval 0.5 nm, and scan speed 100 nm/min. The cuvette was gently shaken prior to all measurements to keep the sample uniformly dispersed. The data collected were background-subtracted using the spectra of GVs in buffer before the GP calculation.

According to the definition by Parasassi and Gratton,^34^ the Laurdan emission spectrum is centered at about 440 nm in the membrane gel phase and at about 490 nm in the membrane liquid crystalline phase. For this reason, it is possible to monitor membrane changes using the so-called Laurdan GP function, defined as
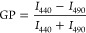
where *I*_440_ and *I*_490_ are the emission intensities at 440 and 490 nm, respectively.

### 2.5 Fluorescence Microscopy Measurements

All fluorescence microscopy experiments were performed depositing aliquots of 250 μL GVs on the microscope-chambered cover glasses (Lab-Tek II Nunc), and the measurements were acquired using a 63× 1.4 oil objective (Leica Microsystems, Wetzlar, Germany) and a scanning frequency of 400 Hz.

#### 2.5.1 Colocalization Experiments

1024 × 1024 pixel resolution images of POPC/POPG and POPC/POPG/Chol GVs after adding CF-TP10 (1.3 μM) were acquired. CF’s fluorescence signal was detected in the range 500–600 nm (λ_exc_ = 470 nm) using a Leica TSC SP5 confocal laser scanning microscope.

#### 2.5.2 Fluorescence Lifetime Imaging Microscopy

256 × 256 pixel FLIM images were collected, before and after peptide addition, in the time domain using the Leica TCS SP5 microscope coupled with a PicoHarp 300 TCSPC Module (PicoQuant, Berlin, Germany).

CF and di-4-ANEPPDHQ fluorescence was acquired using excitation at 470 nm from the pulsed White Light Laser (Leica Microsystem) in the range 500–650 nm. Laurdan fluorescence was acquired under two-photon excitation using λ_exc_ = 780 nm in two channels: 410–460 nm (blue channel) and 480–540 nm (green channel).

### 2.6 FLIM Phasor Plot Analysis and Interpretation

The phasor analysis, described by Digman *et al.*,^35^ was used for FLIM data. Phasor approach is a Fourier domain technique that allows the transformation of the signal in every pixel of the image to a single point called “phasor”. In this representation, all possible single-exponential decays lie on the “universal circle” defined as a semicircle, with radius 1/2, going from point (0, 0), corresponding to τ = ∞, to point (1, 0), corresponding to τ = 0. Instead, complex decays are represented by phasors within the universal circle.

Importantly, given that the phasors follow the vector algebra, it is possible to geometrically resolve the fractions of two fluorescent species (in the simplest case) by the lever rule of vector additions. Indeed, the linear combination of two single-exponential decays components generates phasors within the universal circle, which lie on a straight line joining the phasors of the two single components. The contribution/fraction from one single component to the lifetime is proportional to the distance of the phasor from it. In the phasor plot, it is also possible to select these lifetime distributions using colored cursors and the corresponding pixels will result with the same color of the cursors to the image pixels by which the so-called “lifetime maps” are obtained.

Following previous results in the literature, it may be concluded that if the membrane-sensitive dyes exhibit single-exponential lifetimes, this may arise from homogeneous lipid environments. Instead, multiexponential decays may arise from a mixture of two or more of these lipid environments and lie inside the circle. These considerations allow for recovering information on the subresolution organization of the membranes.^36^

Moreover, with Laurdan, we performed FLIM analysis on measurements in two channels, which provided separate information on the membrane polarity (fluidity, blue channel) and dipolar relaxation (DR) (hydration, green channel).^37^ FLIM data have been processed by the SimFCS software (Laboratory for Fluorescence Dynamics, University of California, Irvine, CA, available at www.lfd.uci.edu). FLIM calibration of the system was performed by measuring the known lifetime of the fluorescein (for di-4-ANEPPDHQ and CF-TP10) that is a single-exponential of 4.0 ns.^38^ For Laurdan measurements, the lifetime calibration was obtained using Alexa 405 considering a single-exponential decay with 3.4 ns lifetime.^39^

## 3 Results

### 3.1 Fluorescence Spectroscopy and Microscopy Analysis of TP10 Interaction with Model Membranes—Effect of Cholesterol

**Figure 1:**
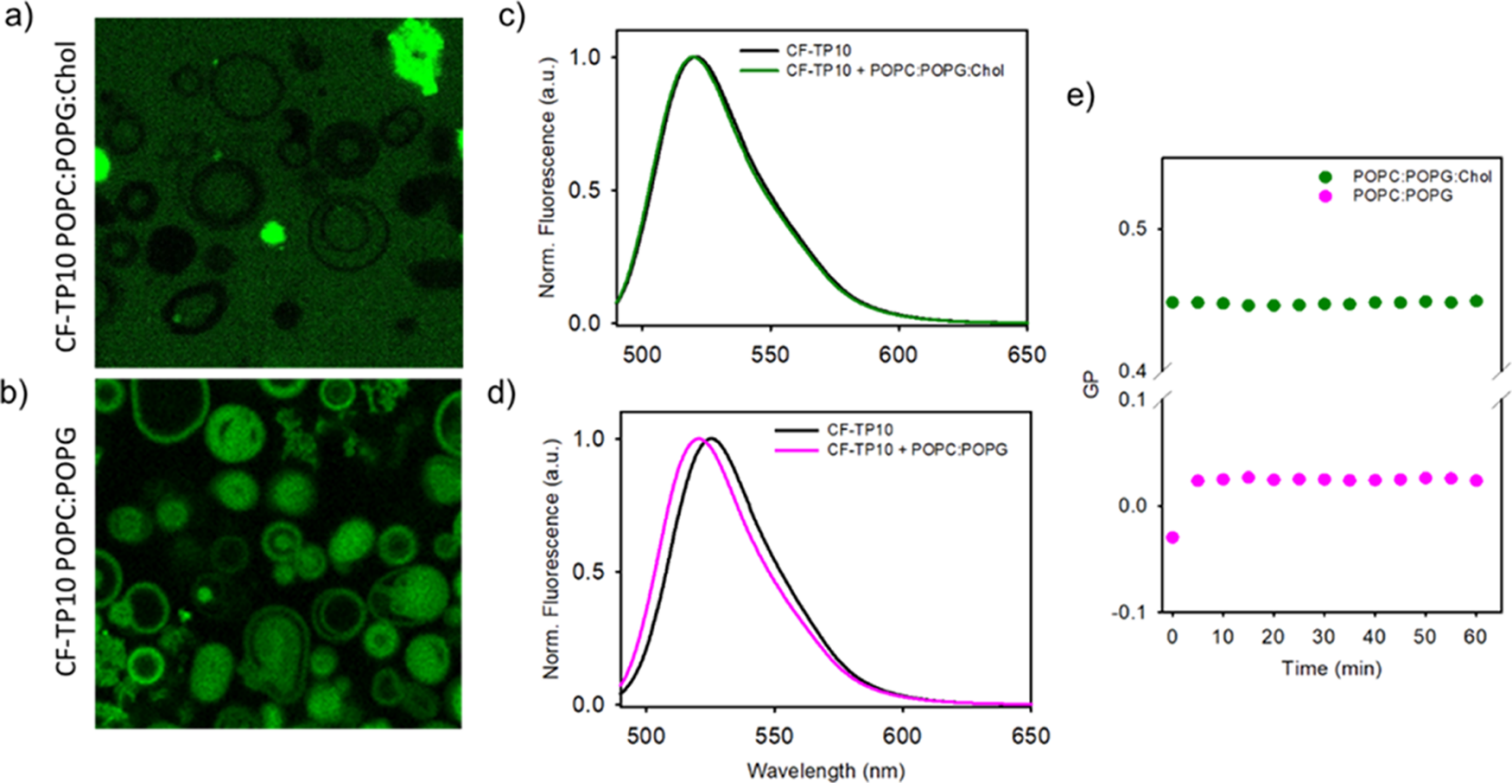
1024 × 1024 pixel representative LSCM measurements of (a) POPC/POPG/Chol GVs and (b) POPC/POPG GVs after the addition of 1.3 μM CF-TP10. The CF fluorescence intensity signal was collected under laser excitation at 470 nm in the range 500–600 nm. Measurements were acquired at equilibrium after 20 h of incubation. In (a), CF fluorescence is found to primarily be distributed in the solution and the GV profiles are identified as dark areas with vanishing fluorescence, indicating that no colocalization exists between CF-TP10 and POPC/POPG membranes. A few brighter structures are identified as CF-TP10 aggregates. In (b), colocalization between CF-TP10 and GV structures is clearly evident, indicating that CF-TP10 is located at the membrane. (c) CF-TP10 normalized fluorescence emission spectra (λ_exc_ = 480 nm), acquired before (black line) and after 20 h (green line) from the addition of CF-TP10 to POPC/POPG/Chol. (d) CF-TP10 normalized fluorescence emission spectra (λ_exc_ = 480 nm), acquired before (black line) and after 20 h (pink line) from the addition of CF-TP10 to POPC/POPG GVs. (e) Time evolution of GP ratios, obtained from the analysis of Laurdan fluorescence spectrum variations, due to the addition of CF-TP10 to POPC/POPG (pink circles) and POPC/POPG/Chol (green circles) GVs. These measurements reveal that the interaction between CF-TP10 and GVs occurs only in membranes where cholesterol is not present.

In Figure 1, we report LSCM and fluorescence spectroscopy measurements for exploring the interaction of the TP10 peptide with POPC/POPG GVs and for verifying the effect of the presence of cholesterol in the membrane structures. Representative 1024 × 1024 pixel images are shown of not labeled POPC/POPG (in a ratio of 2:1) (Figure 1a) and POPC/POPG/Chol (in a ratio of 2:1:2) (Figure 1b) GVs after 20 h of incubation with CF-TP10 (1.3 μM). The CF-TP10 signal is shown in green. The fluorescence emission spectra CF-TP10 obtained in bulk when CF-TP10 is added to GVs containing cholesterol or not are shown in Figure 1c,d, respectively. In particular, Figure 1c reports normalized emission spectra of CF-TP10 (1.3 μM) acquired before (black line) and after (green line) the addition of the peptide to POPC/POPG/Chol GVs, while Figure 1d shows analogous measurements for CF-TP10 before (black line) and after (pink line) the addition to POPC/POPG GVs without cholesterol. In Figure 1e, we also show the time evolution of the GP ratios, obtained from the analysis of Laurdan fluorescence spectrum variations, measured after the addition of TP10 to POPC/POPG (pink circles) and POPC/POPG/Chol (green circles) GVs.

Fluorescence microscopy measurements allowed analysis of sample topology at diffraction-limited resolution as well as allowed mapping the localization of labeled TP10 within the analyzed samples. As can be seen in Figure 1a, green fluorescence is uniformly distributed and no colocalization occurred between CF-TP10 and GVs containing cholesterol. The GV shape appears as black regions. A few bright micron-scale amorphous structures in these samples are identified as CF-TP10 peptide aggregates.

An opposite behavior is found in Figure 1b, where a clear colocalization between the measured green fluorescence signal and GVs is found, indicating that TP10 accumulates at the membrane interface and that, in average, no significant changes in GV shape and morphology are induced by this accumulation. Note that control measurements were carried out via adding free CF to POPC/POPG vesicles, confirming that the dye alone does not localize at the membrane surface under the same experimental conditions (see Supporting Information Figure S1).

Spectral measurements on CF-TP10 fluorescence emission shown in Figure 1c,d aimed at evaluating changes in the CF spectral profile, which may give further information on the possible interactions at the molecular level between CF-TP10 and the model membranes. No significant changes in fluorescence emission spectrum shape are found for POPC/POPG/Chol samples (Figure 1c), while a significant blue shift of the emission maximum from about 530 (black line) to about 520 nm (pink line) is evident for POPC/POPG samples (Figure 1d). The observed shift occurs within the first 5 min after the addition of CF-TP10 to POPC/POPG GVs (see Supporting Information Figure S2), and then, the signal remains stable for at least 24 h. It is well known that changes in fluorescence spectrum shapes and position reflect changes in the fluorophore environment. In particular, fluorescein-based dyes are often used to monitor environmental properties as they are critically sensitive to the pH or the hydrogen-bond character of the environment.^40,41^ Other factors may also affect the spectral properties of these molecules; among these are solvent relaxation phenomena which are often related to spectral shifts and usually reported as a result of changes in the dielectric constant of the surroundings of the dye.^42,43^

The observed shift can therefore be used to obtain a coarse description of the localization/environment of the labeled peptide. Following this idea, observation of CF spectra supplements what is indicated by the LSCM measurements by adding further information at the molecular level. In the presence of cholesterol, TP10 remains in the outer solution not interacting with the membranes, while POPC/POPG GV data indicate a clear change in the environment of CF-TP10, suggesting internalization of the peptide into the membrane or close interaction with it.

Laurdan GP measurements displayed in Figure 1e explore variations in the membrane phase properties^20,44,45^ occurring upon TP10 addition to the GV samples (see Materials and Methods). Laurdan is a gold standard dye used since the ‘70s to monitor changes in membrane organization, reporting membrane packing and fluidity and noninvasively providing dynamic information on membrane heterogeneity at different scales.^44,45^ Laurdan spectral features are thus used to estimate hydration and fluidity changes in the membrane. Indeed, the fluorescence signal of this molecule depends on the physical state of the phase (*e.g.*, local and translational mobility). Changes from a liquid-ordered phase to a liquid-disordered phase induce a shift of the Laurdan spectrum from 440 to 490 nm. The quantification of these spectral changes is usually obtained by considering the GP^33,34,46−48^ which allows one to distinguish the liquid (−0.3 < GP < 0.3) from the gel phase (GP > 0.4)^45^ of the membranes.

The results shown in Figure 1e show that the GP value measured for POPC/POPG/Chol GVs is, as expected and in line with literature values, higher than the one measured for samples in the absence of cholesterol, indicating a higher rigidity and compactness of the membrane.^47,49^ It is not surprising to find that this value remains constant after TP10 addition to cholesterol-containing membranes; this confirms that no modification occurs in the membrane. It is then possible to affirm that no TP10–membrane interactions occur when cholesterol is present.

On the other hand, Laurdan GP in POPC/POPG samples before the addition of TP10 indicates that the membrane is in its liquid phase and results more accessible to the solvent.^45^ After the addition of TP10, the GP value rapidly increased, that is, within the first 5 min. This small but significant change toward a higher GP value can be interpreted as an indication of a progressive change in membrane fluidity toward a more ordered phase. Similar to what was observed in previous studies,^19,20,50^ it is possible to infer that under the tested conditions, TP10 not only accumulates at the surface but also inserts into the membrane causing dehydration and stiffening of the lipid bilayers. This is also in line with the results presented in Figure 1d.

It is worth nothing that the observed TP10 effects do not induce membrane changes in morphology or disruption phenomena. This is in line with a previous study where TP10 translocation phenomena were studied in GVs.^27^ Under the present conditions, after the immediate peptide/membrane interactions, the system remains stable for several hours and no translocation of the peptide is evident. This is not surprising due to the differences in the experimental setup and in the membrane model systems in terms of both lipid composition and lipid organization.^29^ Electrostatic interactions between TP10 positive charges and the anionic head groups of the POPC/POPG lipid vesicles may produce peptide anchoring on the surface.^29,51^

Despite the complexity of the involved mechanisms and of the multiple players, the presence of low cholesterol-containing domains appears to be a key factor for the CPP interactions with the membranes.^52,53^ In the present experiments, we did not detect any sign of interaction, not even adsorption of TP10 to the cholesterol-containing membranes. Analogous results were previously obtained for TP10 and other CPPs and were attributed to the suppression of thermal fluctuations of the membrane.^53^ Also, in more general terms, the results were attributed to the presence of rigid lipid-phases induced by the cholesterol presence.^54−59^ Indeed, as previously suggested, phospholipids above their gel–liquid phase (271 and 274 K^60^ for POPC and POPG, respectively) interact with cholesterol increasing their orientation order and rigidity.^47,49^ Cholesterol presence may also modify the hydrophobic matching between the membrane and peptides, having consequences on their interaction with the lipid bilayer.^61^ The lipid composition and the magnitude of the transmembrane potential may be involved in the membrane selectivity of CPPs, which results in their antimicrobial activity toward bacterial membranes. The evidence that TP10 only interacts with cholesterol-free membranes is an important finding for selective targeting of CPPs to bacterial membranes over eukaryotic ones.^53^ The latter are known to have higher cholesterol content compared to bacterial cell membranes, and thus, eukaryote membranes may be characterized by higher cohesion and stiffness of the lipid bilayer, thus hindering peptide-induced membrane disruption.^58,59,62,63^

Both lipid-ordered and -disordered phases coexist in membranes and may concur in the actual interaction of TP10 and the preferential interaction of CPPs with the disordered phase or in the areas at the edge between different phases. TP10 was suggested to induce bilayer perturbation by causing mass imbalance after adhesion to the outer part of the membrane and/or via a closer interaction possibly resulting in changes in membrane rigidity that may alter membrane curvature, thus causing its disruption.^28^ Its aggregation at the membrane may also occur, as previously reported for others CPPs for which the action was linked to aggregation.^64^

### 3.2 TP10 Adsorption and Insertion into the Membrane: FLIM Analysis on the CF Fluorescence Lifetime

To further explore the occurring interactions between TP10 and negatively charged membranes in the lipid-disordered phase, we decided to focus on the POPC/POPG membrane model described above, exploring the TP10 fate and the effects on the membrane in detail by means of FLIM. FLIM exploits the lifetime properties of fluorescence and presents several advantages with respect to intensity-based methods as it enables combining the sensitivity of fluorescence spectroscopy to image information. This allows mapping of molecular interactions, distinguishing the molecular environment of the fluorophore, and eventually gaining information that is hardly accessible via intensity measurements alone.

These measurements give the possibility to explore simultaneously the peptide localization and the mutual TP10–membrane interaction at a molecular level. To better highlight the changes of physical properties of vesicle membranes and understand the mechanisms of action of the peptide, we explored two different concentration regimes adding TP10 to the membrane at a 300 nM concentration (500:1 lipid/protein ratio) and at a 1.3 μM concentration (115:1 lipid/protein ratio) where significant effects are observed.

TP10 was previously found^65^ to penetrate GVs in a concentration-dependent manner. Moreover, the role of small aggregates was also highlighted. By changing the concentration regime, we explored two largely different conditions where the equilibrium between monomers and possible aggregates was critically shifted toward the aggregated state at a higher concentration. Diffusion-driven interaction was obviously altered and internalization into the membrane possibly favored at higher concentration.

**Figure 2:**
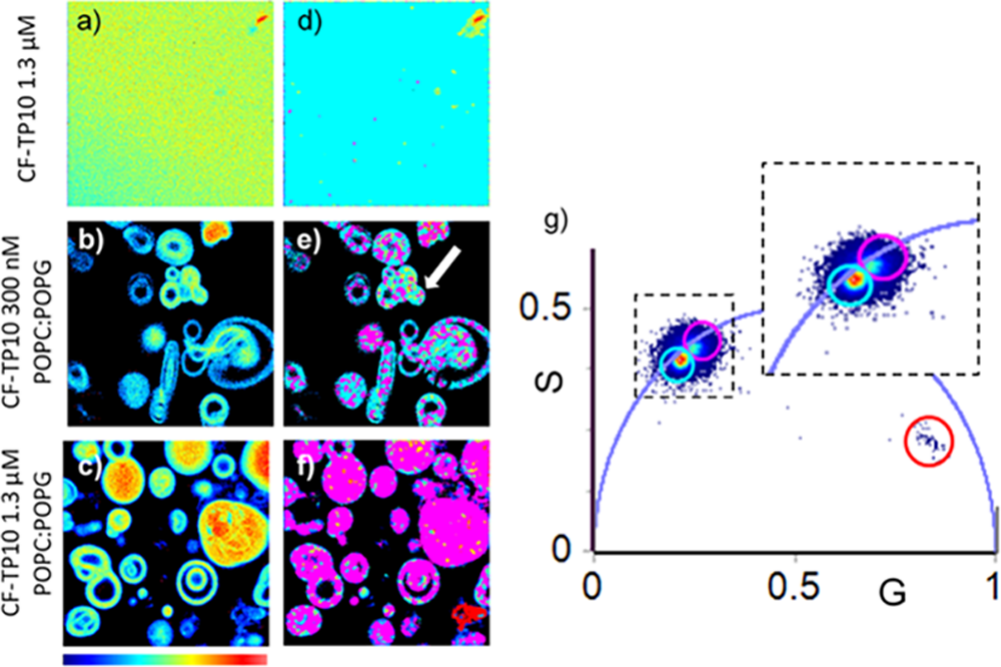
(a–g) Phasor analysis of FLIM measurements on CF-labeled TP10. The signal was acquired under laser excitation at 470 nm and collected in the range 500–650 nm. (a) Intensity map of 1.3 μM CF-TP10 solution in phosphate buffer (20 mM, pH 7) and POPC/POPG GVs 20 h after the addition of (b) 300 nM and (c) 1.3 μM CF-TP10. (d–f) Lifetime maps corresponding to (a–c) measurements colored according to the color code obtained from the phasor plot. (g) Clusters of pixels corresponding to different lifetime distributions identified in the phasor plot and highlighted by colored circular cursors. These pixels are mapped in images (d–f) with corresponding colors. The choice of the size and the position of the circles is arbitrary. A magnification of the region highlighting the area of interest is reported in the dashed line-surrounded inset.

In Figure 2, we show the FLIM results on unstained POPC/POPG GVs acquired 20 h after the addition of the CF-TP10 peptide at 300 nM and 1.3 μM final concentrations. These experiments arise from the observations shown in Figure 1d that reveal CF fluorescence signal sensitivity to TP10 internalization.

FLIM measurements were analyzed by means of the phasor approach.^35^ This fitting-free analysis provides a global view of fluorescence molecule decays at each pixel in the images (see Materials and Methods for details). Phasor analysis is a useful framework to interpret and analyze any possible fluorescence decay without assuming models or performing complex fitting procedures. Moreover, a correspondence exists between the phasor plot points and pixels in the images; this gives the possibility to localize using colored cursors specific pixel clouds in the phasor plot and identify the corresponding pixels in the image.^35^

Each pixel of the intensity images is mapped in a point in the phasor plot corresponding to the measured fluorescence lifetime. Single-exponential lifetimes lie on the so-called “universal circle”. Long lifetimes are localized near the origin (0 on the *x*-axis), while short lifetimes are shifted on the circumference toward the bottom-right insertion with the *x* axis (1 on the *x* axis). As phasors follow the vector algebra, multicomponent-fluorescence decay species result inside the universal circle.^66^

In Figure 2, representative 256 × 256 pixel intensity images of (a) 1.3 μM CF-TP10 in solution and POPC/POPG GV samples 20 h after the addition of (b) 300 nM and (c) 1.3 μM CF-TP10. The corresponding lifetime maps are reported in Figure 2d–f. By selecting lifetime distributions using a circular cursor (cyan and pink), the corresponding pixels are color coded according to the phasor plot in Figure 2g. Specifically, in the phasor plot, two main distinguishable lifetime distributions lying on the universal circle are identified, indicating that CF fluorescence decays can be described as single-lifetime decays (cyan τ_i_ = 3.7 ns and pink τ_f_ = 3.2 ns). A few pixels with shorter lifetime distributions are also observed and selected using a red cursor. Measurements on TP10 in solution in Figure 2a,d reveal, as expected, a uniform intensity and lifetime distribution; all pixels in Figure 2d present a lifetime distribution centered at about 3.7 ns.^67^

The intensity maps in Figure 2b,c, where CF-TP10 was added to GVs at the two concentrations, show that in each sample CF fluorescence was not evenly distributed but that, in average, the samples with a higher concentration of TP10 presented higher intensity. Importantly, as evident in Figure 2e and despite uniform intensity at the single GV level, the measured fluorescence lifetimes are different over a single structure, indicating that CF is experiencing different environments. Pixels exist with lifetimes comparable to the one measured on the sole peptide in solution (cyan); other pixels are characterized by a fluorescence signal with lower lifetime distributions (pink).

The observed decrease in lifetime may be ascribed to water molecule depletion from the environment of the dye or other mechanisms, which increase nonradiative decay processes, for example, the aggregation of the dye bringing the self-quenching mechanisms or the closer lipid membrane interaction of the dye due to the insertion of the peptide in the membrane.^42,67^

In particular, in samples at 300 nM (Figure 2e), results indicate that CF-TP10 mainly interacted with aqueous phases (cyan), without closely interacting with the membrane, and only a few pixels present reduced lifetime (pink). Instead, measurements obtained at 1.3 μM concentration (Figure 2f) reveal that although the GVs maintain their regular morphology almost all pixels are colored in pink, indicating a dense packing of CF-TP10 and/or a higher interaction with membranes. A few pixels highlighted by the red cursor in the phasor plot correspond to amorphous structures that we identify as large peptide aggregates.^67^ The presence of aggregates in samples at higher peptide concentrations possibly suggests that TP10 is added to GV samples in excess with respect to the available interaction sites. It is important to note that observed changes occur right after TP10 addition to the GVs, and analogous measurements acquired within the first hour (see Supporting Information Figure S3) do not present significant changes. Once changes occurred, the sample remains stable at least for 24 h.

### 3.3 TP10 Insertion Effects on the Membrane Structure: FLIM on Membrane-Sensitive Dyes

Taking into account previous data, it is possible to infer that TP10 at high concentration is inserted in the membrane layer. With the aim of focusing on membrane structure changes, fluorescence spectroscopy and FLIM experiments were performed. Two similar dyes, Laurdan and di-4-ANEPPDHQ, were used to label POPC/POPG vesicles. Like Laurdan, di-4-ANEPPDHQ changes its spectral properties depending on membrane organization. Both dyes are reported to sense membranes through analogous mechanisms related to the water molecule accessibility of the lipid bilayer.^32,33,68^ Importantly, for our purposes, these dyes are known to locate at different depths in the membrane as di-4-ANEPPDHQ is sensitive to deeper regions in the hydrophobic core.^68^

**Figure 3:**
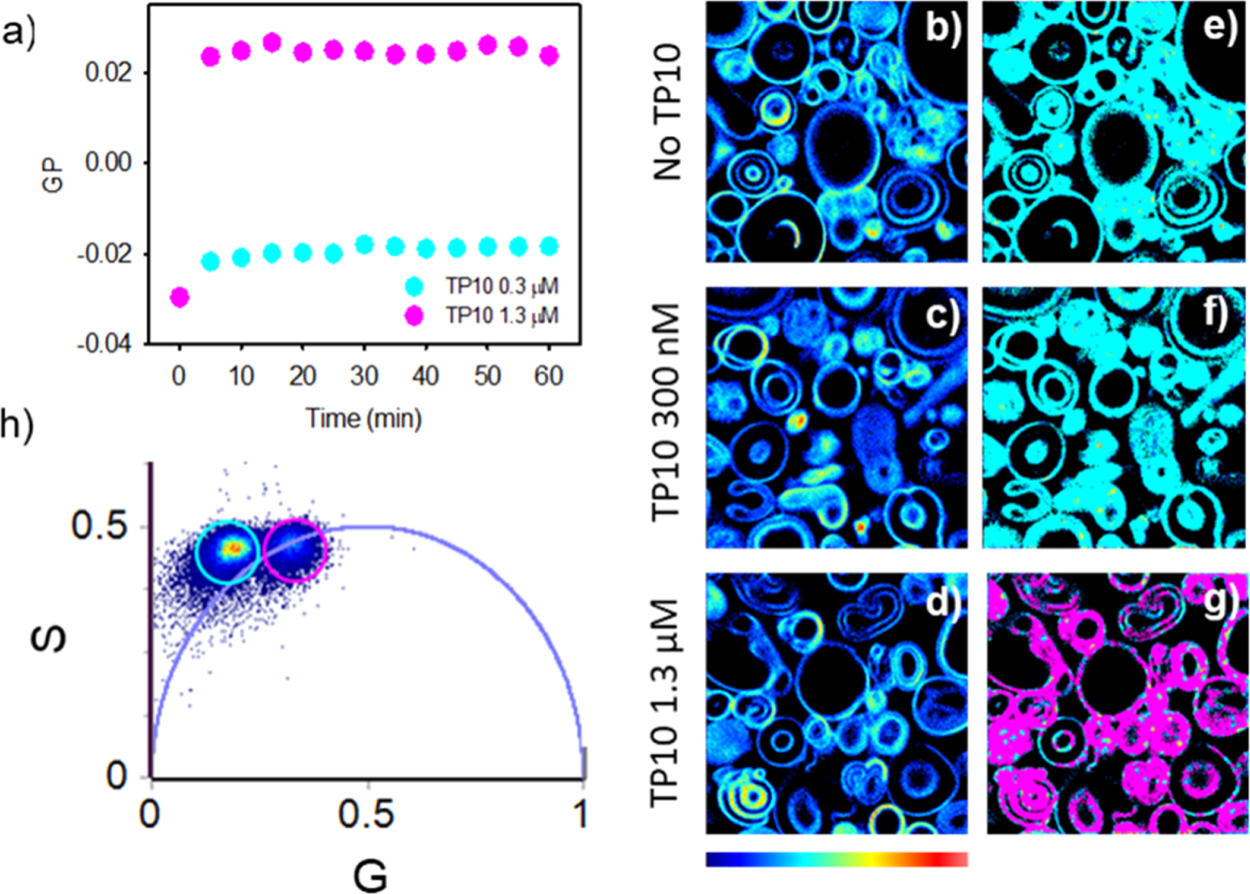
(a) Time evolution of GP ratios obtained from the analysis of Laurdan fluorescence spectrum variations, measured in bulk, after the addition of TP10 at 300 nM (cyan circles) and 1.3 μM (pink circles). (b–h) Phasor analysis of 256 × 256 pixel FLIM measurements on Laurdan in POPC/POPC GVs in the range 410–460 nm, λ_exc_ = 780 nm. Fluorescence intensity images on Laurdan before (b) and after 20 h from the addition of TP10 at 300 nM (c) and 1.3 μM (d). (e–g) Phasor color maps in which each pixel is colored according to the color of the corresponding cursor in the phasor plot. (h) The choice of the size and the position of the cursor is arbitrary.

In Figure 3a, we report Laurdan GP variations as a function of time in bulk experiments performed in a cuvette. We compare the already observed effects measured after adding 1.3 μM TP10 to analogous measurements obtained at 300 nM concentration. Figure 3h shows the phasor analysis of FLIM measurements on Laurdan-stained POPC/POPC GVs acquired in the blue channel (410–460 nm). In Figure 3b–d, 256 × 256 fluorescence intensity images of the analyzed samples are reported. Specifically, in Figure 3b, the signals of GVs before the addition of TP10 are reported together with images acquired after 20 h from the addition of the peptide at 300 nM (c) and 1.3 μM (d). Finally, we show in Figure 3e–g the phasor maps obtained from the same measurements in which each pixel is colored according to the corresponding selection in the phasor plot.

Measurements in Figure 3a take into account the changes in the Laurdan emission spectrum after the addition of TP10. Laurdan is a molecular reporter for membrane organization. Both the number of confined water molecules and their relaxation rates are modified by the physical properties of the membrane. This dye was designed to sense the dielectric environment associated with the solvent properties of its surroundings and the DR of water molecules around the fluorophore dipole in the excited state. These properties make Laurdan fluorescence changes account for both the accessibility of the membrane to water molecules (number of molecules in the Laurdan environment) and to the DR of these few molecules at the membrane interphase which somehow refers to lipid packing.^37,69^

The set of the two effects (which are related to the membrane order) is qualitatively termed as membrane fluidity and is readily reported by GP changes. Data in Figure 3a show a decrease in the fluidity of the membrane, changes occur within 5 min, and there are critically larger changes occurring at higher concentration.

In the phasor plot in Figure 3h, the superimposition of the analysis of all measurements is reported. The phasor analysis of the Laurdan fluorescence lifetime was previously used to separate ordered and disordered phases both in synthetic and in cellular membranes and importantly to evaluate different properties and changes in the membrane structure distinguishing polarity changes and DR allowing pixel by pixel understanding of these two parameters in the membrane.^37^

Two lifetime distributions are evident. The first selected by the cyan cursor is outside the universal circle, which corresponds to pixels (cyan) localized at the membrane in the sample where TP10 is not present and at low TP10 concentration. The other lifetime distribution lies on the universal circle and is highlighted with a pink cursor and only corresponds to pink pixels in the sample where the higher concentration of TP10 is added. Fluorescence-lifetime distributions of Laurdan outside the universal circle can be rationalized considering conditions wherein an excited-state reaction occurs as, for example, fast interconversion processes between Laurdan molecules experiencing a highly dynamic heterogeneous environment.^37^

In the sample at 300 nM, no significant changes occurred for lifetimes measured in this channel with respect to the one measured for untreated samples. Interestingly, the lifetime distribution of Laurdan in GVs after the addition of the peptide at 1.3 μM shifts to a position on the universal circle (the s-coordinate remains constant, while the g-coordinate is reduced). In line with the work of Malacrida and Gratton,^37^ we ascribe these changes to a reduction of membrane hydration levels accompanied by a decrease of DR mechanisms, which refer to the capability of polar solvent molecules in the proximity of the dye to reorient, possibly due to lipid compaction. The final position of the lifetime distribution, single-exponential (lifetime 2.7 ns) indicates a homogeneous lipid environment with coexisting lipid phases.^36^ Also, in this case, it is important to note that analogous measurements acquired within the first hour (see Supporting Information Figure S4) do not present significant changes.

We also applied the same analysis to the green channel (480–540 nm), which confirms inferred changes in DR phenomena (see Supporting Information Figure S5).

We next performed FLIM experiments on the same samples using di-4-ANEPPDHQ. This is with the aim to analyze changes in the inner part of the membranes. The use of FLIM combined with di-4-ANEPPDHQ staining also offers a great contrast in separating the liquid-ordered and liquid-disordered phases. Importantly, for our purposes, di-4-ANEPPDHQ, being aligned with the acyl groups, is located in a more internal and hydrophobic region with respect to Laurdan molecules.^68^ Moreover, this dye contains in its structure two positive charges, which in the present model systems contribute to the reduction of the mobility of the dye within the bilayer.^68^

**Figure 4:**
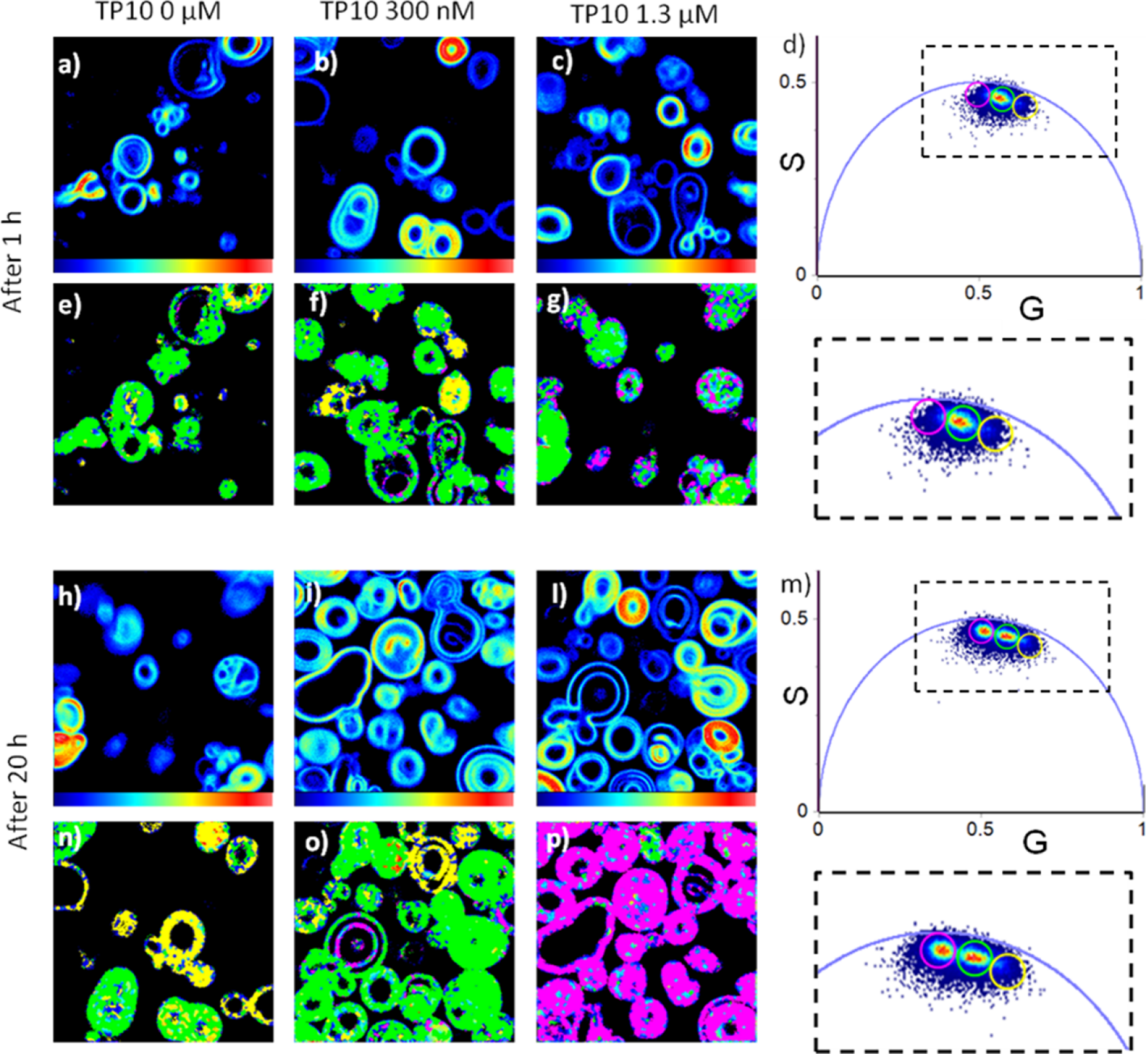
Phasor analysis of 256 × 256 pixel FLIM measurements on di-4-ANEPPDHQ in POPC/POPC GVs after 1 h (a–g) and after 20 h (h–p). Fluorescence intensity images on di-4-ANEPPDHQ before (a–h) and after 1 h (b,c) and 20 h (i,l) from the addition of TP10 at 300 nM (b–i) and 1.3 μM (c–l). (d,m) Phasor plot obtained from measurements (a–c) and (h–l), respectively, where fluorescence-lifetime distributions are highlighted using colored cursors. The magnifications of the regions highlighting area of interests are reported in the dashed line-surrounded inset. (e–g) and (n–p) Phasor color maps in which each pixel is colored according to the color of the corresponding cursor in the phasor plots. The choice of the size and the position of the cursor is arbitrary.

In Figure 4a–c,h–l, we present 256 × 256 pixels of di-4-ANEPPDHQ fluorescence intensity maps in GVs before and after 1 h (a–c) and 20 h (h–l) from TP10 addition at 300 nM and 1.3 μM concentrations. Different from what was observed before, changes on a longer time scale, in the signal reporting for membrane changes under the conditions where TP10 is inserted into the membrane (at 1.3 μM concentration), are found. In this case, measurements acquired 1 h after the addition of the peptide do not superimpose with the ones acquired under stable conditions after incubation for 20 h, indicating that membrane reorganization, following TP10 addition in the presence of this dye, requires a longer time.

In line with previous results, as shown from the intensity images, no significant changes were found in relation to the GV size and shape. In Figure 4d,m, phasors lie inside the universal circle, indicating that di-4-ANEPPDHQ lifetimes, in these conditions, are characterized by nonsingle-exponential decays.^35^ Measurements acquired after 1 h (Figure 4a–d) show a quite broad lifetime distribution reflecting the loosely packed membrane in the fluid phase. No significant changes are observed in samples where the peptide was added at the lower concentration, while a tiny broadening of the distribution toward higher lifetimes is observed in the sample at higher concentration. To visualize this, we used three arbitrary adjacent cursors centered on the straight line, colored in yellow, green, and pink. As can be seen, only in the sample at higher concentration few pink pixels are revealed at the membrane, indicating that the characteristic lifetime of the dye is slightly higher in this region. Characteristic lifetimes vary from shorter (yellow) to longer (pink) as the observed distribution moves counterclockwise in the universal circle. The membrane changes toward the less fluid state.

The same graphical representation is used for phasor analysis of samples incubated with the peptide after 20 h (Figure 4m–p). The phasor plot seems to reveal a broader distribution (two main maxima are identified).

This visualization in three colors at the late stages of incubation shows that the sample, after the addition of 300 nM TP10, is mostly characterized by fluorescence lifetimes selected by the green cursor (green pixels), a reduced number of yellow pixels with respect to the control sample, and a few pink pixels which are characteristic of higher lifetimes are also present. At the higher concentration, the lifetime distribution is shifted toward higher lifetimes so that almost all GVs are selected using the pink cursor, which singles out pixels with more rigid membranes.

The observed results suggest that at lower TP10 concentration the interaction is mostly at the membrane surface and induces small changes in the di-4-ANEPPDHQ environment. When TP10 was added at the high concentration, the peptide inserted deeper into the membrane layer inducing dehydration and stiffening with a consequent increase of di-4-ANEPPDHQ lifetime and membrane reorganization. This requires longer times with respect to changes occurring closer to the water membrane interface monitored by Laurdan.

**Figure 5:**
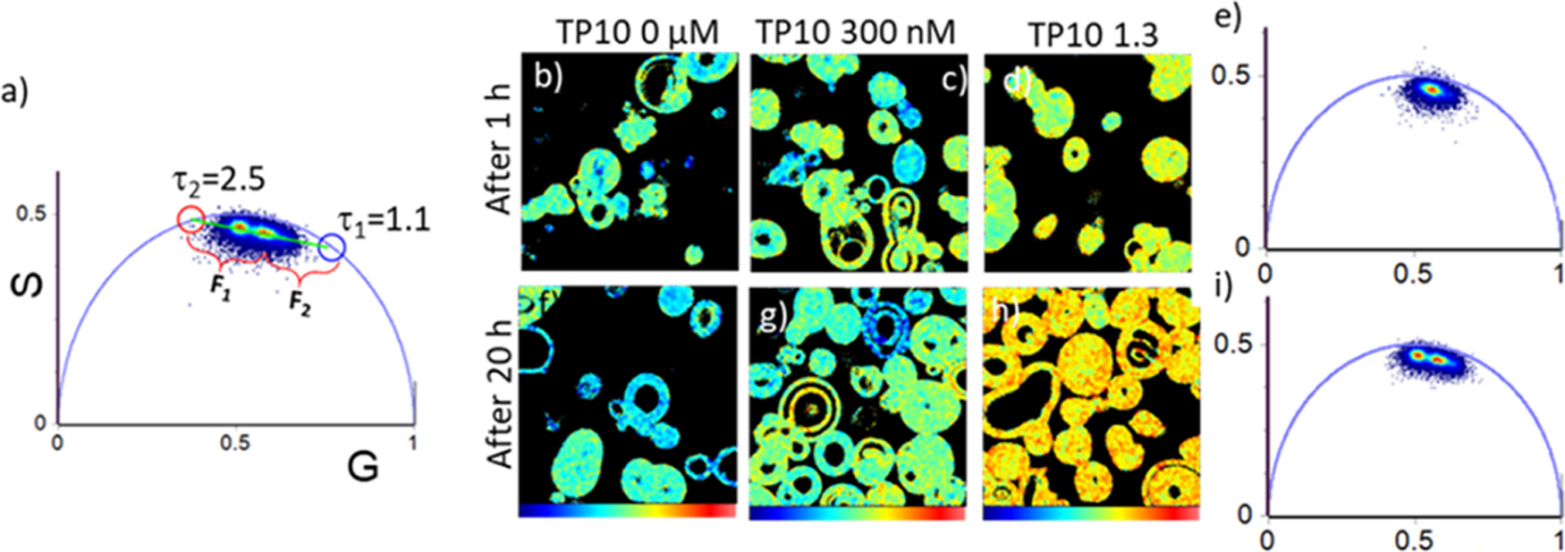
Phasor analysis of 256 × 256 pixel FLIM measurements on di-4-ANEPPDHQ. (a) Phasor plot from the analysis of the FLIM images acquired after 20 h from the addition of TP10 to POPC/POPG labeled with di-4-ANEPPDHQ. The two principal lifetime components, used for the quantitative analysis, are represented by blue and red cursors. Lifetime principal components are τ_1_ = 1.1 ns (blue) and τ_2_ = 2.5 (red). (b–d) Lifetime fraction maps of the FLIM images on di-4-ANEPPDHQ before (b) and after 1 h from the addition of TP10 at 300 nM (c) and 1.3 μM (d). (f–h) Lifetime fraction maps of the FLIM images on di-4-ANEPPDHQ before (f) and after 20 h from the addition of TP10 at 300 nM (g) and 1.3 μM (h). (e,i) Phasor plots from the GVs after 1 h (e) and 20 h (i) from TP10 addition.

In Figure 5, the quantitative analysis of data in Figure 4 is shown. Indeed, looking at the phasor plots, it is possible to draw a straight line where the lifetime distributions lie on (Figure 4d,m), connecting two single-exponential lifetimes of, respectively, τ = 2.5 ns and τ = 1.1 ns. This FLIM analysis is based on the decomposition of the phasor plot data using these two principal lifetime components identified *via* the intersection of the straight line, passing through the lifetime distribution cloud. Following this model, it would be possible to infer that the membrane is characterized by two distinct lipid phases^36^ and not a homogeneous phase of intermediate order. The equilibrium between these phases changes following peptide insertion at different levels in the bilayer.

In Figure 5, we then show the phasor plot where principal components of the decays are highlighted, τ_1_ = 1.1 ns (blue cursor) and τ_2_ = 2.5 ns (red cursor), corresponding to less fluid and more fluid phase, respectively.^36^ Using the two-component model
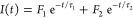


The distance between each point of the cloud in the phasor plot and the single-exponential phasor represents the fraction of each component.^22^ In Figure 5b–d, analysis of the data acquired 1 h after the addition of TP10 is shown, and in Figure 5f–h, the analogous results obtained after the samples have reached the equilibrium at 20 h of incubation. The images are colored in false colors according to the F fraction of the τ component. The scale goes from blue (pure fast component at τ_1_ = 1.1 ns) to red (pure slow component at τ_2_ = 2.5 ns). This scale can be taken into account as a fluidity scale going from less fluid to more rigid membrane configurations.

An increase of di-4-ANEPPDHQ lifetime occurs after the addition of TP10 to POPC/POPG GVs, and this change depends on the peptide concentration and on the incubation time. The fastest decay is dominant in GVs before the peptide addition and when TP10 was added in a concentration of 300 nM (*F*_1_ = 0.44). The average fraction *F*_1_ decreases, increasing the concentration of the added peptide (1.3 μM) being reduced from *F*_1_ = 0.38 (after 1 h) to *F*_1_ = 0.29 (after 20 h). These results suggest that the interactions between the membrane and peptide, when the latter is a high concentration, occur not only at the membrane surface but also in the inner part of the membrane, inducing further reorganization which result in increased rigidity. Electrostatic interactions with POPG charged heads could favor the hold-back of the peptides at the surface, while hydrophobic interactions may tend to drive the peptides into the hydrophobic core of the bilayer. In particular, in the phasor plot relative to the data obtained for di-4-ANEPPDHQ lifetime at a late stage of incubation, two main distributions are clearly evident. In this context, it is possible to infer that the dye experiences two main different environments in terms of membrane fluidity that is induced by TP10 accumulation at the membrane surface with following penetration in the inner parts.

## Conclusions

The analysis of CPP actions, using model membranes, appropriate dyes, and advanced fluorescence techniques, may contribute to a deeper understanding on how membrane-active peptides act in nature under diverse biological conditions. Revealing interactions with details at the molecular level, in terms of both the peptide and membrane structure, may provide suitable information on how membrane-active peptides exert their many functions, on how they may be involved in pathological conditions, and on the molecular strategies developed by bacteria and cells to counteract the action of these molecules.^15,70^ Moreover, disentangling the single role of all these complex multiplayers could be relevant for the design of new synthetic therapeutic agents.

Here, the fate of TP10 and its effects on model membranes are analyzed by means of coupling fluorescence spectroscopy and fluorescence microscopy, which allowed gaining information on the occurring events with molecular details. The TP10 peptide has a bivalent function and depending on experimental conditions, in previous studies, it was found to translocate across the membrane inducing minimal/no perturbation or to act as an antibacterial peptide inducing significant membrane perturbations.^24^ In this study, TP10 is found not to interact with the POPC/POPG membranes enriched with cholesterol despite attractive electrostatic forces due to its positive net charge and the negatively charged lipids in the membranes, suggesting that the presence of cholesterol alters the balance between membrane–solvent and membrane–protein forces which regulate TP10 penetration into the bilayers. In line with the literature, gel–fluid phase coexistence and tighter packing of the lipids, induced by cholesterol, reduce the permeability of the membrane and in the observed conditions completely deplete peptide–membrane interactions.^71^

In the absence of cholesterol, a concentration-dependent interaction between TP10 and the model membrane is found. At lower concentration, only peptide accumulation at the water membrane interface (adsorption) occurs, which does not induce modification in the lateral organization of phospholipids. In these conditions, microsized domains coexist and the fluorescence-labeled TP10 experiences different environments as reported by the lifetime of the CF dye. At higher TP10 concentration, data reveal the insertion of the peptide in the membranes, which correlates with the reduction in membrane fluidity and dehydration measured by the Laurdan dye. Alteration of the bilayer fluidity caused by interaction with the peptide can lead to instability of the membrane structure and to an enhanced membrane weakness that can be the origin of altered cells and microbial behavior. Interestingly, the analysis of di-4-ANEPPDHQ, which is positively charged and located in a more internal region with respect to Laurdan, reveals further details and shows that a slower membrane reorganization occurs, leading to the formation of micron-sized domains with overall reduction in membrane fluidity. The observed modifications in the membrane structure appear to imply changes in the subresolution domain heterogeneity as suggested by Laurdan measurements and do not hinder the morphology of the membranes.

The presented results show that the parallel use of multiple targeted molecular reporters and FLIM/phasor analysis may highlight diverging aspects of such multifaceted complex phenomena as peptide–membrane interaction at a single liposome level and in three dimensions, allowing the possibility of following dynamic events in real time without sample manipulation. In this context, the analysis through the phasor approach, not based on calculations or nonlinear fitting, also allows an intuitive and graphical representation of FLIM data.^35^

FLIM experiments provide image analysis tools which have great potential to disentangle spatially heterogeneous phenomena. This method can be used to parallel highly informative bulk methods such as NMR spectroscopy^72^ and dynamic light scattering,^73^ which can be used to provide useful insights into the membrane–peptide interaction. The conformational changes occurring in the membranes and peptides can be monitored by circular dichroism and infrared analysis,^74^ while complementary information at the nanoscale as the morphology of protein layers at interfaces can be revealed by atomic force microscopy^75^ and thin-film X-ray diffraction.^76^ Furthermore, surface and structural information about proteins in situ and in real time could be provided by the noninvasive and label-free sum-frequency generation spectroscopy.^77^

## Supporting Information Available

CF FLIM measurements, 1.3 μM CF-TP10 fluorescence emission spectra, CF-TP10 FLIM measurements after 1 h, Laurdan FLIM measurements after 1 h blue channel, and Laurdan FLIM measurements after 1 h green channel (PDF)

## References

[ref1] Galdiero S.; Falanga A.; Cantisani M.; Vitiello M.; Morelli G.; Galdiero M. Peptide-lipid interactions: Experiments and applications. Int. J. Mol. Sci. 2013, 14, 18758–18789. 10.3390/ijms140918758. 24036440 PMC3794806

[ref2] Bucciantini M.; Rigacci S.; Stefani M. Amyloid aggregation: Role of biological membranes and the aggregate-membrane system. J. Phys. Chem. Lett. 2014, 5, 517–527. 10.1021/jz4024354. 26276603

[ref3] Stefani M. Biochemical and biophysical features of both oligomer/fibril and cell membrane in amyloid cytotoxicity. FEBS J. 2010, 277, 4602–4613. 10.1111/j.1742-4658.2010.07889.x. 20977664

[ref4] Butterfield S. M.; Lashuel H. A. Amyloidogenic protein-membrane interactions: mechanistic insight from model systems. Angew. Chem., Int. Ed. Engl. 2010, 49, 5628–5654. 10.1002/anie.200906670. 20623810

[ref5] Pieri L.; Madiona K.; Bousset L.; Melki R. Fibrillar α-Synuclein and Huntingtin Exon 1 Assemblies Are Toxic to the Cells. Biophys. J. 2012, 102, 2894–2905. 10.1016/j.bpj.2012.04.050. 22735540 PMC3379023

[ref6] D’Angelo F.; Vignaud H.; Di Martino J.; Benedicte S.; Devin A.; Cullin C.; Marchal C. A yeast model for amyloid-β aggregation exemplifies the role of membrane trafficking and PICALM in cytotoxicity. Dis. Models Mech. 2013, 6, 206–216. 10.1242/dmm.010108. PMC3529352 22888099

[ref7] Shai Y. Mode of action of membrane active antimicrobial peptides. Biopolymers 2002, 66, 236–248. 10.1002/bip.10260. 12491537

[ref8] Rahnamaeian M.; Cytryńska M.; Zdybicka-Barabas A.; Vilcinskas A. The functional interaction between abaecin and pore-forming peptides indicates a general mechanism of antibacterial potentiation. Peptides 2016, 78, 17–23. 10.1016/j.peptides.2016.01.016. 26845197

[ref9] Mahlapuu M.; Håkansson J.; Ringstad L.; Björn C. Antimicrobial peptides: An emerging category of therapeutic agents. Front. Cell. Infect. Microbiol. 2016, 6, 194 10.3389/fcimb.2016.00194. 28083516 PMC5186781

[ref10] Yeaman M. R.; Yount N. Y. Mechanisms of antimicrobial peptide action and resistance. Pharmacol. Rev. 2003, 55, 27–55. 10.1124/pr.55.1.2. 12615953

[ref11] Milanesi L.; Sheynis T.; Xue W.-F.; Orlova E. V.; Hellewell A. L.; Jelinek R.; Hewitt E. W.; Radford S. E.; Saibil H. R. Direct three-dimensional visualization of membrane disruption by amyloid fibrils. Proc. Natl. Acad. Sci. U. S. A. 2012, 109, 20455–20460. 10.1073/pnas.1206325109. 23184970 PMC3528594

[ref12] Zemel A.; Ben-Shaul A.; May S. Perturbation of a lipid membrane by amphipathic peptides and its role in pore formation. Eur. Biophys. J. 2005, 34, 230–242. 10.1007/s00249-004-0445-9. 15619088

[ref13] Bobone S.; Piazzon A.; Orioni B.; Pedersen J. Z.; Nan Y. H.; Hahm K.-S.; Shin S. Y.; Stella L. The thin line between cell-penetrating and antimicrobial peptides: The case of Pep-1 and Pep-1-K. J. Pept. Sci. 2011, 17, 335–341. 10.1002/psc.1340. 21294230

[ref14] Sani M.-A.; Separovic F. How Membrane-Active Peptides Get into Lipid Membranes. Acc. Chem. Res. 2016, 49, 1130–1138. 10.1021/acs.accounts.6b00074. 27187572

[ref15] Peschel A. How do bacteria resist human antimicrobial peptides?. Trends Microbiol. 2002, 10, 179–186. 10.1016/s0966-842x(02)02333-8. 11912025

[ref16] Li J.; Chikindas M. L.; Ludescher R. D.; Montville T. J. Temperature- and surfactant-induced membrane modifications that alter Listeria monocytogenes nisin sensitivity by different mechanisms. Appl. Environ. Microbiol. 2002, 68, 5904–5910. 10.1128/aem.68.12.5904-5910.2002. 12450809 PMC134382

[ref17] Mishra N. N.; Bayer A. S.; Tran T. T.; Shamoo Y.; Mileykovskaya E.; Dowhan W.; Guan Z.; Arias C. A. Daptomycin resistance in enterococci is associated with distinct alterations of cell membrane phospholipid content. PLoS One 2012, 7, e43958 10.1371/journal.pone.0043958. 22952824 PMC3428275

[ref18] Fernández-Pérez E. J.; Sepúlveda F. J.; Peters C.; Bascuñán D.; Riffo-Lepe N. O.; González-Sanmiguel J.; Sánchez S. A.; Peoples R. W.; Vicente B.; Aguayo L. G. Effect of cholesterol on membrane fluidity and association of Aβ oligomers and subsequent neuronal damage: A Double-Edged Sword. Front. Aging Neurosci. 2018, 10, 226 10.3389/fnagi.2018.002.26. 30123122 PMC6085471

[ref19] Van Maarschalkerweerd A.; Vetri V.; Langkilde A. E.; Foderà V.; Vestergaard B. Protein/lipid coaggregates are formed during α-synuclein-induced disruption of lipid bilayers. Biomacromolecules 2014, 15, 3643–3654. 10.1021/bm500937p. 25210839

[ref20] Van Maarschalkerweerd A.; Vetri V.; Vestergaard B. Cholesterol facilitates interactions between α-synuclein oligomers and charge-neutral membranes. FEBS Lett. 2015, 589, 2661–2667. 10.1016/j.febslet.2015.08.013. 26297828

[ref21] Sameni S.; Malacrida L.; Tan Z.; Digman M. A. Alteration in Fluidity of Cell Plasma Membrane in Huntington Disease Revealed by Spectral Phasor Analysis. Sci. Rep. 2018, 8, 734 10.1038/s41598-018-19160-0. 29335600 PMC5768877

[ref22] Vetri V.; Ossato G.; Militello V.; Digman M. A.; Leone M.; Gratton E. Fluctuation methods to study protein aggregation in live cells: Concanavalin a oligomers formation. Biophys. J. 2011, 100, 774–783. 10.1016/j.bpj.2010.11.089. 21281593 PMC3030242

[ref23] Saar K.; Lindgren M.; Hansen M.; Eiríksdóttir E.; Jiang Y.; Rosenthal-Aizman K.; Sassian M.; Langel Ü. Cell-penetrating peptides: A comparative membrane toxicity study. Anal. Biochem. 2005, 345, 55–65. 10.1016/j.ab.2005.07.033. 16137634

[ref24] Nekhotiaeva N.; Elmquist A.; Rajarao G. K.; Hällbrink M.; Langel U.; Good L. Cell entry and antimicrobial properties of eukaryotic cell-penetrating peptides. FASEB J. 2004, 18, 394–396. 10.1096/fj.03-0449fje. 14656995

[ref25] Moghal M. M. R.; Islam M. Z.; Hossain F.; Saha S. K.; Yamazaki M. Role of Membrane Potential on Entry of Cell-Penetrating Peptide Transportan 10 into Single Vesicles. Biophys. J. 2019, 118, 57–69. 10.1016/j.bpj.2019.11.012. 31810658 PMC6950768

[ref26] Kristensen M.; Birch D.; Mørck Nielsen H. Applications and challenges for use of cell-penetrating peptides as delivery vectors for peptide and protein cargos. Int. J. Mol. Sci. 2016, 17, 185 10.3390/ijms17020185. PMC4783919 26840305

[ref27] Islam M. Z.; Ariyama H.; Alam J. M.; Yamazaki M. Entry of cell-penetrating peptide transportan 10 into a single vesicle by translocating across lipid membrane and its induced pores. Biochemistry 2014, 53, 386–396. 10.1021/bi401406p. 24397335

[ref28] Yandek L. E.; Pokorny A.; Florén A.; Knoelke K.; Langel Ü.; Almeida P. F. F. Mechanism of the cell-penetrating peptide transportan 10 permeation of lipid bilayers. Biophys. J. 2007, 92, 2434–2444. 10.1529/biophysj.106.100198. 17218466 PMC1864827

[ref29] Bárány-Wallje E.; Gaur J.; Lundberg P.; Langel Ü.; Gräslund A. Differential membrane perturbation caused by the cell penetrating peptide Tp10 depending on attached cargo. FEBS Lett. 2007, 581, 2389–2393. 10.1016/j.febslet.2007.04.046. 17485081

[ref30] Stulz A.; Vogt A.; Saar J. S.; Akil L.; Lienkamp K.; Hoernke M. Quantified Membrane Permeabilization Indicates the Lipid Selectivity of Membrane-Active Antimicrobials. Langmuir 2019, 35, 16366–16376. 10.1021/acs.langmuir.9b01849. 31710807

[ref31] Sanders M. R.; Clifton L. A.; Frazier R. A.; Green R. J. Role of Lipid Composition on the Interaction between a Tryptophan-Rich Protein and Model Bacterial Membranes. Langmuir 2016, 32, 2050–2057. 10.1021/acs.langmuir.5b04628. 26813886

[ref32] Amaro M.; Reina F.; Hof M.; Eggeling C.; Sezgin E. Laurdan and Di-4-ANEPPDHQ probe different properties of the membrane. J. Phys. D: Appl. Phys. 2017, 50, 134004 10.1088/1361-6463/aa5dbc. 29449744 PMC5802044

[ref33] Owen D. M.; Rentero C.; Magenau A.; Abu-Siniyeh A.; Gaus K. Quantitative imaging of membrane lipid order in cells and organisms. Nat. Protoc. 2012, 7, 24–35. 10.1038/nprot.2011.419. 22157973

[ref34] Parasassi T.; De Stasio G.; d’Ubaldo A.; Gratton E. Phase fluctuation in phospholipid membranes revealed by Laurdan fluorescence. Biophys. J. 1990, 57, 1179–1186. 10.1016/s0006-3495(90)82637-0. 2393703 PMC1280828

[ref35] Digman M. A.; Caiolfa V. R.; Zamai M.; Gratton E. The phasor approach to fluorescence lifetime imaging analysis. Biophys. J. 2008, 94, L14–L16. 10.1529/biophysj.107.120154. 17981902 PMC2157251

[ref36] Owen D. M.; Williamson D. J.; Magenau A.; Gaus K. Sub-resolution lipid domains exist in the plasma membrane and regulate protein diffusion and distribution. Nat. Commun. 2012, 3, 1256 10.1038/ncomms2273. 23212385

[ref37] Malacrida L.; Gratton E. Laurdan fluorescence and phasor plots reveal the effects of a H2O2 bolus in NIH-3T3 fibroblast membranes dynamics and hydration. Free Radic. Biol. Med. 2018, 128, 144–156. 10.1016/j.freeradbiomed.2018.06.004. 29885356 PMC6175669

[ref38] Data Tables. Fluorescence Lifetime Standards. ISS.

[ref39] Van Zanten C.; Melnikau D.; Ryder A. G. Effects of Viscosity and Refractive Index on the Emission and Diffusion Properties of Alexa Fluor 405 Using Fluorescence Correlation and Lifetime Spectroscopies. J. Fluoresc. 2021, 31, 835–845. 10.1007/s10895-021-02719-y. 33740150

[ref40] Klonis N.; Clayton A. H. A.; Voss E. W.; Sawyer W. H. Spectral Properties of Fluorescein in Solvent-Water Mixtures: Applications as a Probe of Hydrogen Bonding Environments in Biological Systems. Photochem. Photobiol. 1998, 67, 500–510. 10.1111/j.1751-1097.1998.tb09085.x. 9613235

[ref41] Arrabito G.; Cavaleri F.; Porchetta A.; Ricci F.; Vetri V.; Leone M.; Pignataro B. Printing Life-Inspired Subcellular Scale Compartments with Autonomous Molecularly Crowded Confinement. Adv. Biosyst. 2019, 3, 1900023 10.1002/adbi.201900023. 32648672

[ref42] Lakowicz J. R. Principles of Fluorescence Spectroscopy; Springer, 2006.

[ref43] Demchenko A. P.; Mély Y.; Duportail G.; Klymchenko A. S. Monitoring biophysical properties of lipid membranes by environment-sensitive fluorescent probes. Biophys. J. 2009, 96, 3461–3470. 10.1016/j.bpj.2009.02.012. 19413953 PMC2711402

[ref44] Parasassi T.; Krasnowska E. K.; Bagatolli L.; Gratton E. Laurdan and Prodan as Polarity-Sensitive Fluorescent Membrane Probes. J. Fluoresc. 1998, 8, 365–373. 10.1023/a:1020528716621.

[ref45] Sanchez S. A.; Tricerri M. A.; Gunther G.; Gratton E. Laurdan Generalized Polarization: from cuvette to microscope. Mod. Res. Educ.Top. Microsc. 2007, 2, 1007–1014.

[ref46] Bagatolli L. A.; Gratton E. Two-photon fluorescence microscopy observation of shape changes at the phase transition in phospholipid giant unilamellar vesicles. Biophys. J. 1999, 77, 2090–2101. 10.1016/s0006-3495(99)77050-5. 10512829 PMC1300490

[ref47] Harris F. M.; Best K. B.; Bell J. D. Use of laurdan fluorescence intensity and polarization to distinguish between changes in membrane fluidity and phospholipid order. Biochim. Biophys. Acta Biomembr. 2002, 1565, 123–128. 10.1016/s0005-2736(02)00514-x. 12225860

[ref48] Sánchez S. A.; Tricerri M. A.; Ossato G.; Gratton E. Lipid packing determines protein-membrane interactions: Challenges for apolipoprotein A-I and high density lipoproteins. Biochim. Biophys. Acta Biomembr. 2010, 1798, 1399–1408. 10.1016/j.bbamem.2010.03.019. PMC2883020 20347719

[ref49] Fidorra M.; Duelund L.; Leidy C.; Simonsen A. C.; Bagatolli L. A. Absence of fluid-ordered/fluid-disordered phase coexistence in ceramide/POPC mixtures containing cholesterol. Biophys. J. 2006, 90, 4437–4451. 10.1529/biophysj.105.077107. 16565051 PMC1471871

[ref50] Rao E.; Foderà V.; Leone M.; Vetri V. Direct observation of alpha-lactalbumin, adsorption and incorporation into lipid membrane and formation of lipid/protein hybrid structures. Biochim. Biophys. Acta Gen. Subj. 2019, 1863, 784–794. 10.1016/j.bbagen.2019.02.005. 30742952

[ref51] Hincha D. K.; Crowe J. H. The lytic activity of the bee venom peptide melittin is strongly reduced by the presence of negatively charged phospholipids or chloroplast galactolipids in the membranes of phosphatidylcholine large unilamellar vesicles. Biochim. Biophys. Acta Biomembr. 1996, 1284, 162–170. 10.1016/s0005-2736(96)00122-8. 8914580

[ref52] Lorents A.; Säälik P.; Langel Ü.; Pooga M. Arginine-Rich Cell-Penetrating Peptides Require Nucleolin and Cholesterol-Poor Subdomains for Translocation across Membranes. Bioconjugate Chem. 2018, 29, 1168–1177. 10.1021/acs.bioconjchem.7b00805. 29510042

[ref53] Islam M. Z.; Sharmin S.; Levadnyy V.; Alam Shibly S. U.; Yamazaki M. Effects of Mechanical Properties of Lipid Bilayers on the Entry of Cell-Penetrating Peptides into Single Vesicles. Langmuir 2017, 33, 2433–2443. 10.1021/acs.langmuir.6b03111. 28166411

[ref54] Hasan M.; Moghal M. M. R.; Saha S. K.; Yamazaki M. The role of membrane tension in the action of antimicrobial peptides and cell-penetrating peptides in biomembranes. Biophys. Rev. 2019, 11, 431–448. 10.1007/s12551-019-00542-1. 31093936 PMC6557934

[ref55] Arsov Z.; Nemec M.; Schara M.; Johansson H.; Langel Ü.; Zorko M. Cholesterol prevents interaction of the cell-penetrating peptide transportan with model lipid membranes. J. Pept. Sci. 2008, 14, 1303–1308. 10.1002/psc.1062. 18683276

[ref56] Crosio M. A.; Via M. A.; Cámara C. I.; Mangiarotti A.; Del Pópolo M. G.; Wilke N. Interaction of a polyarginine peptide with membranes of different mechanical properties. Biomolecules 2019, 9, 625 10.3390/biom9100625. PMC6843195 31635304

[ref57] Golfetto O.; Hinde E.; Gratton E. Laurdan fluorescence lifetime discriminates cholesterol content from changes in fluidity in living cell membranes. Biophys. J. 2013, 104, 1238–1247. 10.1016/j.bpj.2012.12.057. 23528083 PMC3602759

[ref58] Epand R.; Ramamoorthy A.; Epand R. Membrane Lipid Composition and the Interaction of Pardaxin: The Role of Cholesterol. Protein Pept. Lett. 2006, 13, 1–5. 10.2174/0929866510602010001. 16454662

[ref59] Hallock K. J.; Lee D.-K.; Omnaas J.; Mosberg H. I.; Ramamoorthy A. Membrane composition determines Pardaxin’s mechanism of lipid bilayer disruption. Biophys. J. 2002, 83, 1004–1013. 10.1016/s0006-3495(02)75226-0. 12124282 PMC1302204

[ref60] Wiedmann T.; Salmon A.; Wong V. Phase behavior of mixtures of DPPC and POPG. Biochim. Biophys. Acta, Lipids Lipid Metab. 1993, 1167, 114–120. 10.1016/0005-2760(93)90150-8. 8466937

[ref61] Henderson J. M.; Iyengar N. S.; Lam K. L. H.; Maldonado E.; Suwatthee T.; Roy I.; Waring A. J.; Lee K. Y. C. Beyond electrostatics: Antimicrobial peptide selectivity and the influence of cholesterol-mediated fluidity and lipid chain length on protegrin-1 activity. Biochim. Biophys. Acta Biomembr. 2019, 1861, 182977 10.1016/j.bbamem.2019.04.011. 31077677

[ref62] Matsuzaki K.; Sugishita K.; Fujii N.; Miyajima K. Molecular Basis for Membrane Selectivity of an Antimicrobial Peptide, Magainin 2. Biochemistry 1995, 34, 3423–3429. 10.1021/bi00010a034. 7533538

[ref63] Matsuzaki K.; Murase O.; Fujii N.; Miyajima K. Translocation of a Channel-Forming Antimicrobial Peptide, Magainin 2, Across Lipid Bilayers by Forming a Pore. Biochemistry 1995, 34, 6521–6526. 10.1021/bi00019a033. 7538786

[ref64] Wadhwani P.; Reichert J.; Bürck J.; Ulrich A. S. Antimicrobial and cell-penetrating peptides induce lipid vesicle fusion by folding and aggregation. Eur. Biophys. J. 2012, 41, 177–187. 10.1007/s00249-011-0771-7. 22080286 PMC3269571

[ref65] Ruczyński J.; Rusiecka I.; Turecka K.; et al. Transportan 10 improves the pharmacokinetics and pharmacodynamics of vancomycin. Sci. Rep. 2019, 9, 3247 10.1038/s41598-019-40103-w. 30824786 PMC6397271

[ref66] Stringari C.; Cinquin A.; Cinquin O.; Digman M. A.; Donovan P. J.; Gratton E. Phasor approach to fluorescence lifetime microscopy distinguishes different metabolic states of germ cells in a live tissue. Proc. Natl. Acad. Sci. U. S. A. 2011, 108, 13582–13587. 10.1073/pnas.1108161108. 21808026 PMC3158156

[ref67] Chen R. F.; Knutson J. R. Mechanism of fluorescence concentration quenching of carboxyfluorescein in liposomes: Energy transfer to nonfluorescent dimers. Anal. Biochem. 1988, 172, 61–77. 10.1016/0003-2697(88)90412-5. 3189776

[ref68] Dinic J.; Biverståhl H.; Mäler L.; Parmryd I. Laurdan and di-4-ANEPPDHQ do not respond to membrane-inserted peptides and are good probes for lipid packing. Biochim. Biophys. Acta Biomembr. 2011, 1808, 298–306. 10.1016/j.bbamem.2010.10.002. 20937246

[ref69] Malacrida L.; Jameson D. M.; Gratton E. A multidimensional phasor approach reveals Laurdan photophysics in NIH-3T3 cell membranes. Sci. Rep. 2017, 7, 9215 10.1038/s41598-017-08564-z. 28835608 PMC5569084

[ref70] Xiong Y. Q.; Mukhopadhyay K.; Yeaman M. R.; Adler-Moore J.; Bayer A. S. Functional interrelationships between cell membrane and cell wall in antimicrobial peptide-mediated killing of Staphylococcus aureus. Antimicrob. Agents Chemother. 2005, 49, 3114–3121. 10.1128/aac.49.8.3114-3121.2005. 16048912 PMC1196293

[ref71] Mason A. J.; Marquette A.; Bechinger B. Zwitterionic Phospholipids and Sterols Modulate Antimicrobial Peptide-Induced Membrane Destabilization. Biophys. J. 2007, 93, 4289–4299. 10.1529/biophysj.107.116681. 17766347 PMC2098721

[ref72] Sattler M.; Schleucher J.; Griesinger C. Heteronuclear multidimensional NMR experiments for the structure determination of proteins in solution employing pulsed field gradients. Prog. Nucl. Magn. Reson. Spectrosc. 1999, 34, 93–158. 10.1016/s0079-6565(98)00025-9.

[ref73] Carpenter D. K.; Berne B. J. Dynamic Light Scattering with Applications to Chemistry, Biology, and Physics (Berne, Bruce J.; Pecora, Robert). J. Chem. Educ. 1977, 54, A430 10.1021/ed054pa430.1.

[ref74] Sreerama N.; Woody R. W. Estimation of protein secondary structure from circular dichroism spectra: Comparison of contin, selcon, and CDSSTR methods with an expanded reference set. Anal. Biochem. 2000, 287, 252–260. 10.1006/abio.2000.4880. 11112271

[ref75] Rief M.; Gautel M.; Oesterhelt F.; Fernandez J. M.; Gaub H. E. Reversible unfolding of individual titin immunoglobulin domains by AFM. Science 1997, 276, 1109–1112. 10.1126/science.276.5315.1109. 9148804

[ref76] Zhou Y.; Hu N.; Zeng Y.; Rusling J. F. Heme Protein–Clay Films:Direct Electrochemistry and Electrochemical Catalysis. Langmuir 2002, 18, 211–219. 10.1021/la010834a.

[ref77] Wang Z.; Fu L.; Ma G.; Yan E. C. Y. Broad-Bandwidth Chiral Sum Frequency Generation Spectroscopy for Probing the Kinetics of Proteins at Interfaces. Langmuir 2015, 31, 11384–11398. 10.1021/acs.langmuir.5b02100. 26196215 PMC4625692

