# Supplementary material for: Peptide–Membrane Interactions Monitored by Fluorescence Lifetime Imaging: A Study Case of Transportan 10
Source: Langmuir. 2021 Oct 29;37(44):13148–59. doi: 10.1021/acs.langmuir.1c02392 (PMC8582253; doi:10.1021/acs.langmuir.1c02392)
Supplement: Supplementary file 1 — la1c02392_si_001.pdf [file la1c02392_si_001.pdf]

SUPPORTING INFORMATION

Peptide-membrane interactions monitored by fluorescence lifetime imaging: a study case of Transportan 10

Sara Anselmo[1], Giuseppe Sancataldo[1] and Hanne Mørck Nielsen[2], Vito Foderà[2] and Valeria Vetri[1*]

[1]Dipartimento di Fisica e Chimica – Emilio Segré, Università degli Studi di Palermo, Viale delle Scienze ed. 18, 90128 Palermo, Italy
[2]Department of Pharmacy, University of Copenhagen, Universitetsparken 2, 2100 Copenhagen, Denmark

*Corresponding author: valeria.vetri@unipa.it

Figure S1. Carboxyfluorescein FLIM measurements

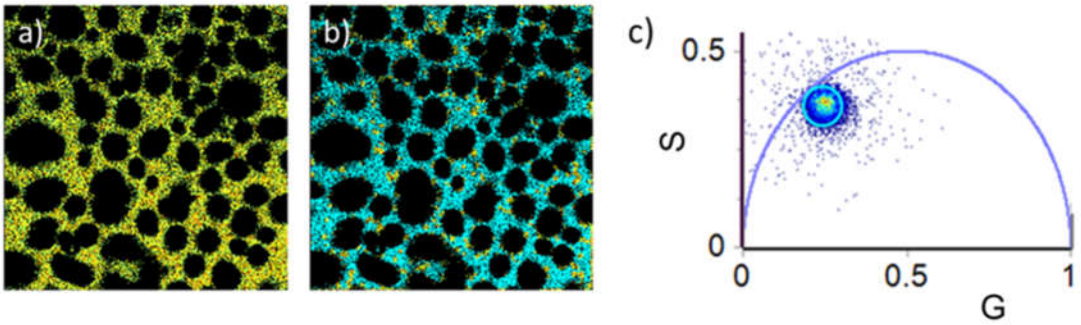

Fig. S1. 256 × 256 pixel FLIM measurements of 1.3 μM Carboxyfluorescein (CF) excited at λ_{exc}=470 nm and collected in the range 500-650 nm when added to POPC:POPG GVs, as a control for experiments in fig. 2. (a) Intensity map and (b) phasor color map: each pixel in (b) is colored according to the color of the corresponding circle in the phasor plot illustrated in (c) measurements reveal no differences between this sample and the one in the absence of vesicles.

Figure S2. 1.3μM CF-TP10 fluorescence emission spectra

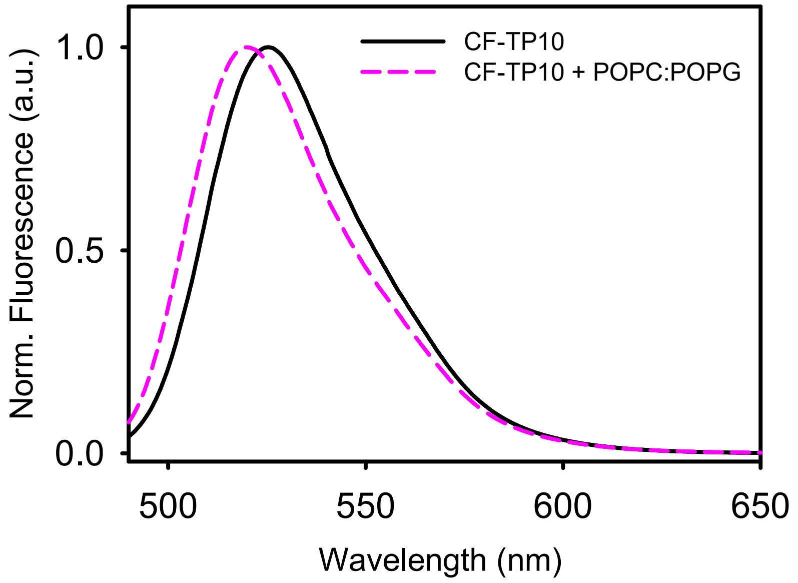

Fig. S2. 1.3 μM CF-TP10 normalised fluorescence emission spectra (λex = 480 nm), acquired before (black line) and after 5 min (pink dashed line) from the addition of CF-TP10 to POPC:POPG GVs sample.

Figure S3. CF-TP10 FLIM measurements after one hour

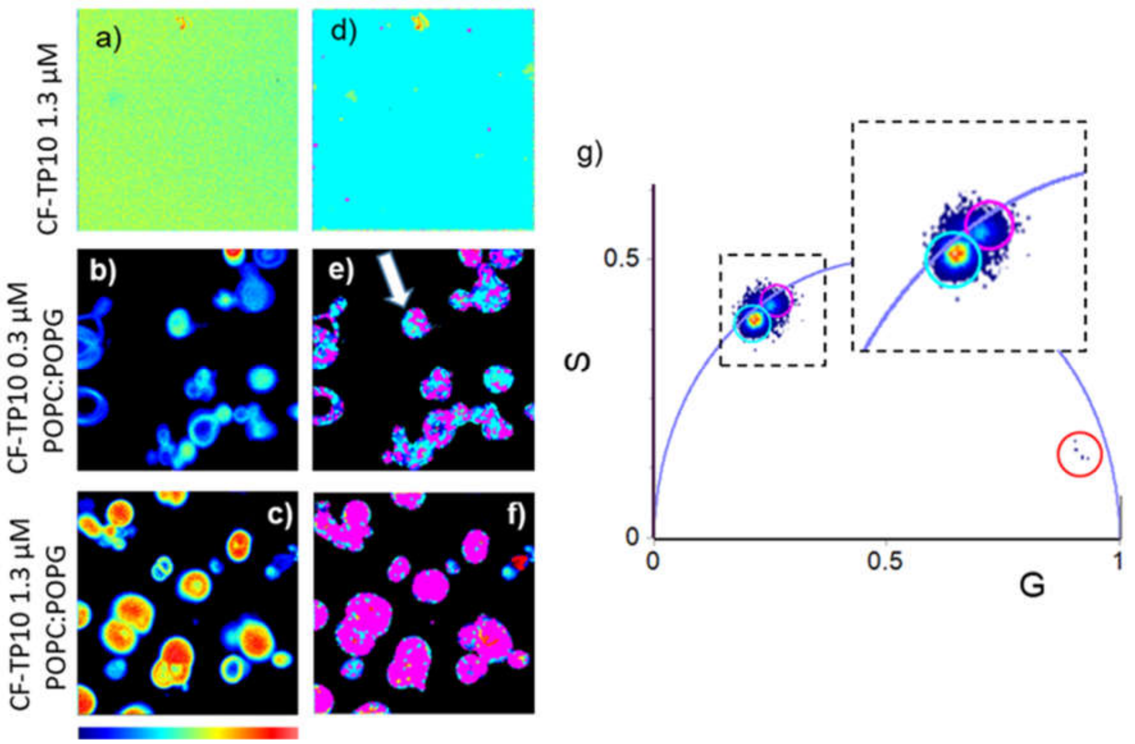

Fig. S3. Phasor analysis of FLIM measurements on CF-TP10 signal under laser excitation at 470 nm in the range 500-650 nm. (a) Intensity map of 1.3 µM CF-TP10 solution in phosphate buffer 20 mM pH 7 (b) POPC:POPG GVs 1 hour after the addition of 300 nM and (c) 1.3 µM CF-TP10 (d-f). Lifetime map corresponding to (a-c) measurements coloured according to the colour code obtained from the phasor plot (g). Cluster of pixel corresponding to different lifetime distribution have been identified in the phasor plot and are highlighted by colored circular cursors: these pixels are mapped in images (d-f) with corresponding colours. The choice of the size and the position of the circles is arbitrary. A magnification of the region highlighting area of interest is reported in the dashed lines surrounded inset.

Figure S4. Laurdan FLIM measurements after one hour- blue channel

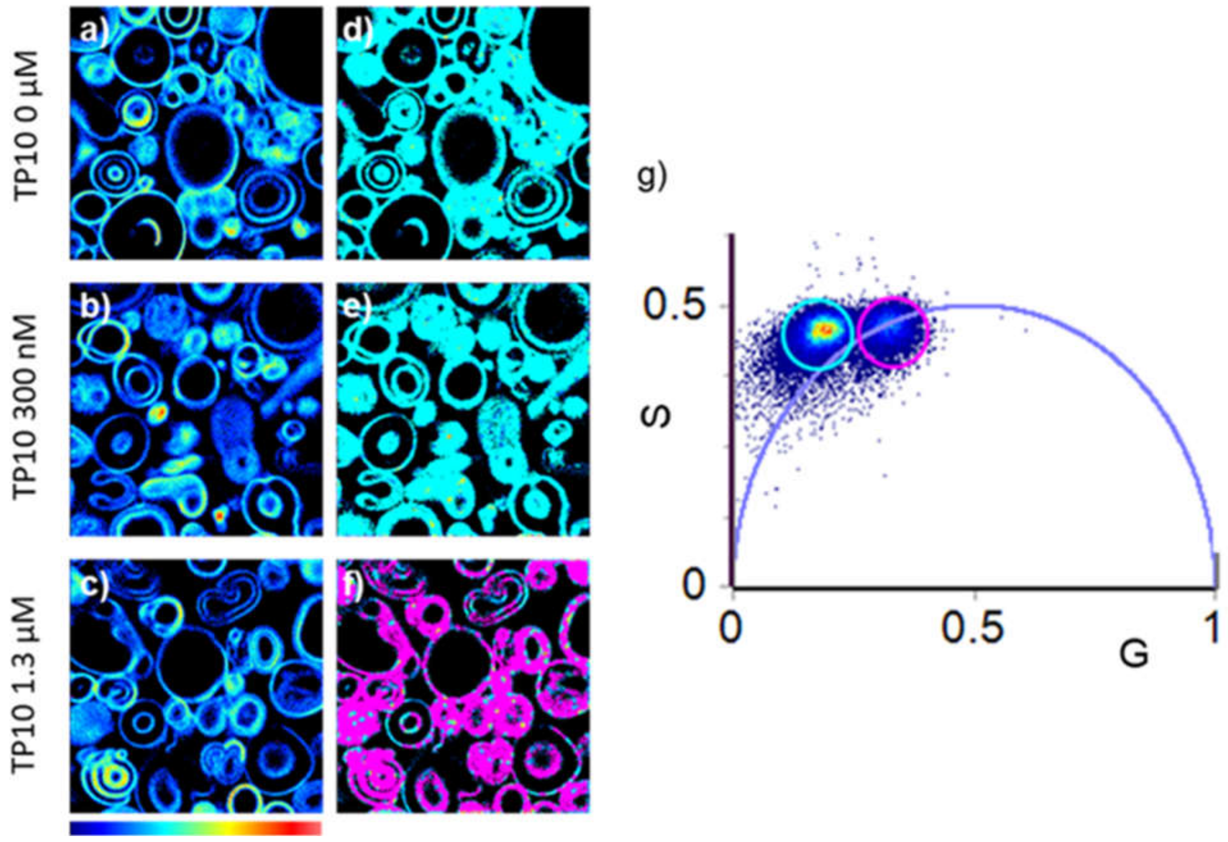

Fig. S4. Phasor analysis of 256 × 256 pixel FLIM measurements on Laurdan in POPC:POPC GVs in the range 410-460nm, λ_{exc}= 780 nm. Fluorescence intensity images on Laurdan before (a) and after one hour from the addition of TP10 300 nM (b) and 1.3 µM (c). (g) Phasor plot obtained from measurements (a-c). (d-f) Phasor color maps in which each pixel is colored according to the color of the corresponding cursor in the phasor plot. The choice of the size and the position of the cursor is arbitrary.

Figure S5. Laurdan FLIM measurements after one hour- green channel

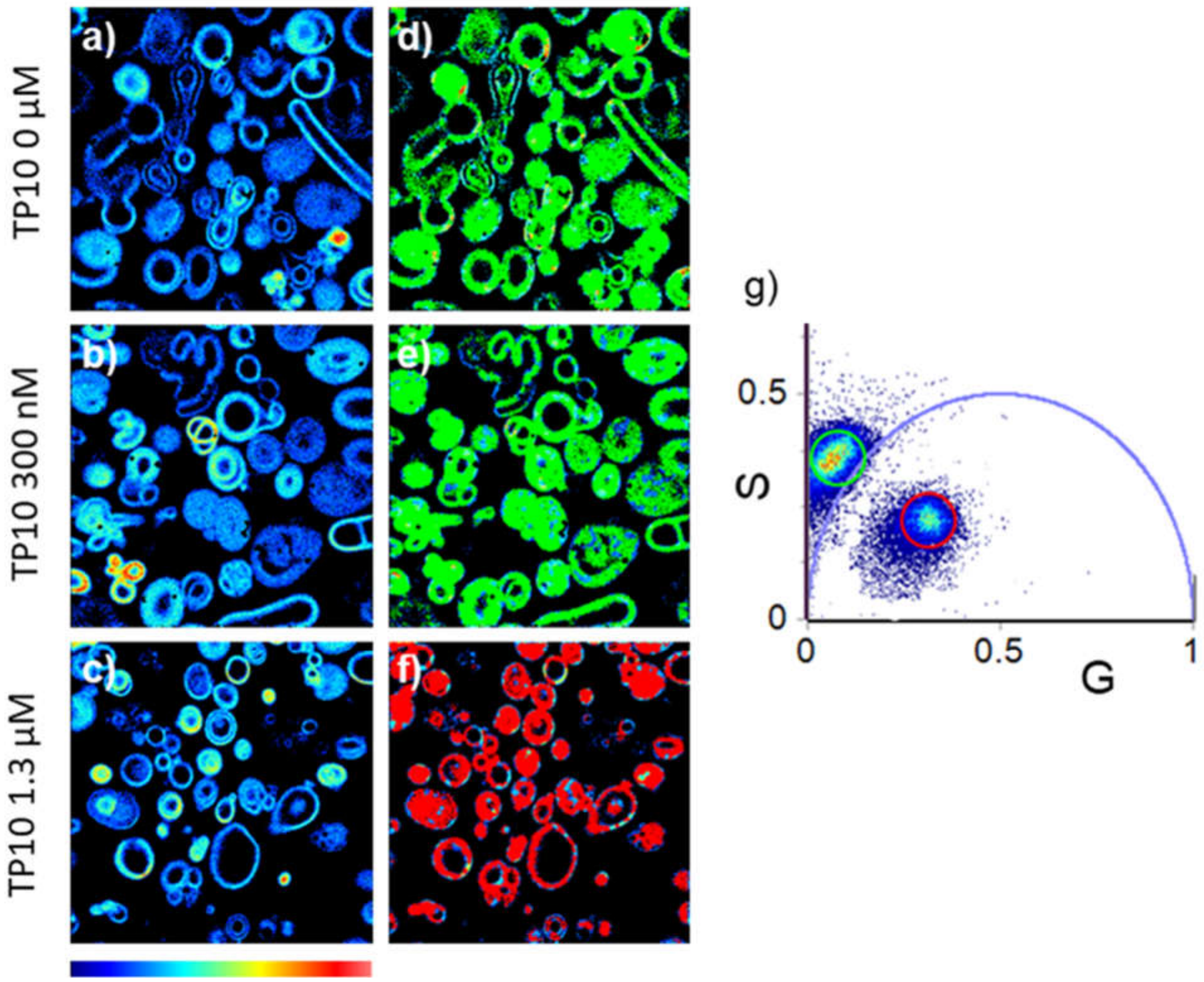

Fig. S5. Phasor analysis of 256 × 256 pixel FLIM measurements on Laurdan in POPC:POPC GVs in the range 480-540nm, λ_{exc}= 780nm. Fluorescence intensity images on Laurdan before (a) and after one hour from the addition of TP10 300 nM (b) and 1.3 μM (c). (g) Phasor plot obtained from measurements (a-c). (d-f) Phasor color maps in which each pixel is colored according to the color of the corresponding cursor in the phasor plot. The choice of the size and the position of the cursor is arbitrary. In the green channel is not possible highlight differences in polarity (fluidity). At opposite, the addition of TP10 1.3 μM induce a significant decrease of the dipolar relaxation (hydration). Analogous measurements acquired after 20 h do not present significant changes.
